# Diagnostic determinants of acid-fast bacilli culture positivity in miliary TB

**DOI:** 10.5588/ijtldopen.25.0579

**Published:** 2026-04-13

**Authors:** J. Yang, M. Choi, K.S. Yi, Y.J. Lee, E.C. Kim, G. Min, M. Kim, E. Park, J.Y. Cho, Y.M. Shin, K.H. Choe, K.M. Lee, B. Yang, S.-H. Kim

**Affiliations:** 1 Division of Pulmonary and Critical Care Medicine, Department of Internal Medicine, Chungbuk National University Hospital, Chungbuk National University College of Medicine, Cheongju, South Korea;; 2 The Medical Artificial Intelligence Center, Chungbuk National University Hospital, Cheongju, South Korea;; 3 Department of Radiology, Chungbuk National University Hospital, Chungbuk National University College of Medicine, Cheongju, South Korea;; 4 Department of Biochemistry, College of Medicine, Chungbuk National University, Cheongju, South Korea;; 5 Academic Cooperation Foundation, Chungbuk National University Industry, Cheongju, South Korea;; 6 Department of Clinical Trials Center, Biomedical Research Institute, Chungbuk National University Hospital, Cheongju, South Korea.

**Keywords:** tuberculosis, cavitary lesions, computed tomography, diagnostic predictors

Dear Editor,

Miliary TB is a severe form of TB caused by *Mycobacterium tuberculosis*, and is characterised by haematogenous dissemination and the presence of multiple miliary nodules on chest imaging.^1,2^ It can affect individuals regardless of their immune status and, if not diagnosed and treated promptly, is associated with high mortality. The diagnosis of miliary TB remains challenging owing to its nonspecific clinical presentation and diverse radiological manifestations.^3^ Sputum-based tests, including smear microscopy and culture, can aid *M. tuberculosis* detection; however, their sensitivity is variable and generally lower in miliary TB, complicating diagnosis. Definitive diagnosis relies on acid-fast bacilli (AFB) smear and culture, nucleic acid amplification tests (NAATs), and histopathological examination. However, AFB smears have low sensitivity, culture is time-consuming, and NAAT results are not consistently positive.^3,4^ Although microbiological culture remains the gold standard for diagnosing pulmonary TB, its prolonged turnaround time (2–8 weeks) limits its utility in miliary TB, where early diagnosis is crucial. Despite this, in smear-negative miliary TB cases, culture is usually the only definitive diagnostic tool.^5^ Given these diagnostic challenges, identifying the differences between culture-positive and culture-negative cases is essential for developing effective diagnostic strategies for miliary TB. Therefore, we aimed to investigate these factors to enhance diagnostic strategies and support timely clinical decision-making in miliary TB.

We reviewed the medical records of patients with TB who were assigned the A19 diagnostic code between January 2015 and December 2023. In the Korean Standard Classification of Diseases, which is based on the ICD-10 system, the A19 code corresponds to miliary TB. Seventy-one patients were initially identified using the A19 code. After independent CT review by two pulmonologists, 18 patients were excluded, yielding a final cohort of 53 patients. Demographic and comorbidity data were collected through a retrospective review of the medical records. One radiologist and two pulmonologists reviewed all CT images in a blinded manner, and decisions were reached based on the consensus. Miliary TB was diagnosed in patients with a miliary nodule pattern on CT if *M. tuberculosis* was microbiologically identified, granulomatous inflammation was histologically confirmed, or clinical and radiological findings improved following empirical anti-TB therapy. Sputum AFB smears and cultures were prepared using standard methods.^4^ Conventional drug susceptibility testing was performed using the absolute concentration method on Lowenstein–Jensen medium at the Korean Institute of Tuberculosis.^5^ We compared continuous variables using the Mann–Whitney *U* test and categorical variables using Pearson’s χ² test or Fisher’s exact test. To identify factors associated with AFB culture positivity in miliary TB, we performed multivariable logistic regression analysis. The covariates included age, sex, body mass index, history of TB, comorbidities, AFB smear status, and radiological features. This study was approved by the Institutional Review Board of Chungbuk National University Hospital (IRB Approval No. 2025-08-003).

The baseline characteristics of the 53 patients with miliary TB are presented in Supplementary Data Table S1. Patients were classified as culture-negative (n = 16, 30.2%) or culture-positive (n = 37, 69.8%) based on AFB culture results. Baseline characteristics did not differ significantly between the two groups. Supplementary Data Table S2 summarises microbiological and treatment outcomes, with no significant differences observed in AFB smear results, NAAT positivity, or treatment outcomes between the two groups. Bronchoscopy was performed in 26 patients (49.1%). Bronchoscopic AFB culture positivity was significantly higher in the culture-positive group (81.3% vs. 0%, *P* = 0.005). Biopsies were obtained in 15 patients (51.7%), more frequently in the culture-negative than in the culture-positive group (80.0% vs. 37.0%, *P* = 0.051), suggesting a trend toward significance. Chest CT findings are summarised in Supplementary Data Table S3. Among the CT findings, only cavitary lesions differed significantly between the two groups (0% vs. 34.3%, *P* = 0.010). Multivariate logistic regression identified the presence of cavitary lesions as the only factor independently associated with AFB culture positivity (adjusted odds ratio [aOR]: 16.39, 95% confidence interval [CI]: 1.14–3815.70; *P* = 0.038) (see Table). Representative chest CT images are shown in Supplementary Data Figure S1, demonstrating cavitary lesions in culture-positive patients and their absence in culture-negative patients.

**Table.** Logistic regression analysis of factors associated with AFB culture in miliary TB.

	Univariate analysis	*P* value	Multivariate analysis	*P* value
	Unadjusted OR		aOR (95% CI)	
Age	0.97 (0.93–1.01)	0.088	0.99 (0.94–1.03)	0.637
BMI	0.97 (0.82–1.16)	0.761	1.03 (0.85–1.25)	0.761
Previous history of TB	0.62 (0.09–4.11)	0.618	0.17 (0.00–2.51)	0.213
Diabetes mellitus	2.90 (0.32–26.33)	0.343	3.26 (0.55–34.08)	0.201
Sputum AFB smear	1.63 (0.30–8.89)	0.570	1.16 (0.10–16.28)	0.905
TBc PCR	4.11 (0.79–21.48)	0.094	1.56 (0.64–4.28)	0.336
Bronchoscopy	0.46 (0.14–1.52)	0.202	1.02 (0.23–4.42)	0.976
Cavity	16.2 (1.89–2133.01)	0.006	16.39 (1.14–3815.70)	0.038

OR = odds ratio; aOR = adjusted odds ratio; CI = confidence interval; BMI = body mass index; AFB = acid-fast bacillus; TBc = tuberculosis complex; PCR = polymerase chain reaction.

This study investigated the diagnostic determinants of AFB culture positivity in miliary TB. The overall culture positivity rate was 69.8%. Cavitary lesions were more frequent in the culture-positive group, and multivariate analysis confirmed that the presence of cavities was independently associated with AFB culture positivity. AFB culture positivity rates in sputum samples from patients with miliary TB have been reported to vary widely, with a range of 14.9%–83.8%.^3,6–9^ In this study, a relatively high positivity rate of 69.8% was observed. Such variations may be attributable to differences in patient characteristics, testing methods, and timing of specimen collection, which should be carefully considered when interpreting AFB culture results.

Radiological findings showed that the presence of cavitary lesions was significantly higher in the AFB culture-positive group, which is a crucial feature of our study that has not been previously reported. Miliary TB spreads systemically via lymphatic and haematogenous routes and manifests as numerous uniformly sized nodules (approximately 2 mm). Additionally, centrilobular and tree-in-bud patterns may be observed.^10,11^ In contrast, pulmonary TB cavities form when the central necrotic area of granulomatous lung abscesses caused by *M. tuberculosis* connects to the airways.^12^ Owing to the different pathophysiological mechanisms of pulmonary TB, cavitary lesions are rare in miliary TB and have primarily been reported in patients who are immunocompromised, with limited case reports in previous studies.^13,14^ However, in this study, we revealed that the simultaneous presence of the miliary TB pattern and cavitary lesions in immunocompetent patients suggests that cavities may also play a crucial diagnostic role in miliary TB.

Another strength of this study is the comprehensive analysis of factors associated with AFB culture positivity in patients with miliary TB, incorporating clinical, microbiological, and radiological variables. In multivariate logistic regression, the presence of cavitary lesions was independently and significantly associated with AFB culture positivity, whereas other variables did not show statistically significant associations (aOR: 16.39, 95% CI: 1.14–3815.70; *P* = 0.038). To date, few studies have comprehensively investigated the predictors of culture positivity in miliary TB, and they lack direct and systematic analyses of this topic. Some review articles have suggested that immunocompromised conditions, such as HIV infection, immunosuppressive therapy, or organ transplantation, may be associated with increased culture positivity; however, such conclusions remain speculative.^3^ Our findings provide valuable insights for predicting culture positivity in miliary TB, in which smear-negative results are common. Notably, the presence of cavitary lesions strongly suggests miliary TB and highlights the need for more active microbiological investigations.

This study has several limitations. First, its retrospective design and small sample size limit generalisability. In particular, the wide CI observed for the aOR of cavitary lesions indicates limited statistical precision, likely due to the small number of events. This should be considered when interpreting the strength and reliability of this association. Second, extra-pulmonary involvement and immunocompromised status were rare in this cohort, which may restrict applicability to broader populations. Third, AFB culture positivity from bronchial washings was lower than previously reported,^15^ although yields improved when biopsy specimens were included, underscoring the importance of bronchoscopy as a diagnostic tool.

In conclusion, cavitary lesions independently predict AFB culture positivity in miliary TB, reinforcing the role of imaging in guiding timely diagnosis and management.

## Supplementary Material
