# Supplementary material for: Diagnostic determinants of acid-fast bacilli culture positivity in miliary TB
Source: IJTLD Open. 2026 Apr 13;3(4):269–71. doi: 10.5588/ijtldopen.25.0579 (PMC13080311; doi:10.5588/ijtldopen.25.0579)

Supplementary Table 1. Baseline characteristics of miliary TB patients

	All patients (n = 53)	Culture negative miliary TB (n = 16)	Culture positive miliary TB (n=37)	P-value
Age, years	72 (51-79)	75 (68-80)	70 (45-79)	0.200
Sex, male	28 (52.8)	10 (62.5)	18 (48.6)	0.354
BMI, kg/m^2	19.5 (18.7-22.8)	20.3 (18.8-22.6)	19.4 (18.1-22.9)	0.490
Smoking history				0.650
Never-smoker	29 (54.7)	8 (50.0)	21 (56.8)	
Current- or ex-smoker	24 (45.3)	8 (50.0)	16 (43.2)	
Previous history of tuberculosis	5 (9.4)	2 (12.5)	3 (8.1)	0.632
Comorbidities				
Diabetes Mellitus	7 (13.2)	1 (6.3)	6 (16.2)	0.661
Hypertension	23 (43.4)	9 (56.3)	14 (37.8)	0.214
COPD	2 (3.8)	1 (6.3)	1 (2.7)	0.517
Asthma	1 (1.9)	0	1 (2.7)	1.000
Cardiovascular disease	7 (13.2)	2 (12.5)	5 (13.5)	1.000
Neurologic disease	9 (17.0)	4 (25.0)	5 (13.5)	0.427
Chronic liver disease	2 (3.8)	1 (6.3)	1 (2.7)	0.517
Chronic kidney disease	5 (9.4)	2 (12.5)	3 (8.1)	0.632
Malignancy	7 (13.2)	4 (25.0)	3 (8.1)	0.179
HIV	0			
Rheumatoid disease	4 (7.5)	3 (18.8)	1 (2.7)	0.077
Involvement of other organs				0..258
CNS	1 (1.9)	0	1 (2.7)	
Bone	6 (11.3)	1 (6.3)	5 (13.5)	
Urinary tract	1 (1.9)	0	1 (2.7)	

Data are presented as the median (interquartile range) or number (%).

Abbreviations: TB, tuberculosis; BMI, body mass index; COPD, chronic obstructive pulmonary disease; HIV, Human immunodeficiency virus

Supplementary Table 2. Microbiology and treatment in miliary TB patients

Microbiology	All patients (n = 53)	Culture negative miliary TB (n = 16)	Culture positive miliary TB (n=37)	P-value
Sputum AFB smear				0.706
Negative	44 (83.0)	14 (87.5)	30 (81.1)	
Positive	9 (17.0)	2 (12.5)	7 (18.9)	
Sputum AFB culture				<0.001
Negative	16 (34.0)	16 (100)	2 (5.4)	
Positive	35 (66.0)	0	35 (94.6)	
Sputum NAAT	Tested/ Total patients			
TBc PCR	15/46 (32.6)	2/14 (14.3)	13/32 (40.6)	0.099
Xpert	11/24 (45.8)	2/9 (22.2)	9/15 (60.0)	0.105
Bronchoscopy	26 (49.1)	10 (62.5)	16 (43.2)	0.198
AFB smear				1.000
Negative	25 (96.2)	10 (100)	15 (93.8)	
Positive	1 (3.8)	0	1 (6.2)	
AFB culture				0.005
Negative	13 (50.0)	10 (100)	3 (18.7)	
Positive	13 (50.0)	0	13 (81.3)	
NAAT				
Tbc PCR	8/26 (30.8)	1 (10.0)	7 (43.8)	0.099
Biopsy	14 (53.8)	8 (80.0)	6 (37.5)	0.051
Chronic granulmatous inflammation or tuberculosis	10/14 (71.4)	6/8 (75.0)	4/6 (66.7)	1.000
Positive Tbc PCR in tissue	4/14 (28.6)	1/8 (12.5)	3/6 (50.0)	0.194
Treatment duration	182 (45-270)	181 (119-252)	183 (31-270)	0.764
Treatment results				0.646
Cure	15 (28.3)	5 (31.3)	10 (27.0)	
Treatment completion	15 (28.3)	6 (37.5)	9 (24.3)	
Treatment failure	0			
Death	12 (22.6)	3 (18.8)	9 (24.3)	
Follow up loss	11 (20.8)	2 (12.5)	9 (24.3)	

Data are presented as the median (interquartile range) or number (%).

Abbreviations: TB, tuberculosis; AFB, acid-fast bacillus; PCR, polymerase chain reaction; NAAT, nucleic acid amplification test

Supplementary Table 3. Chest CT findings for miliary TB patients

Radiologic features		Culture negative miliary TB (n = 16)	Culture positive miliary TB (n=37)	P-value
Random distribution	53 (100)	16 (100)	37 (100)	
Centrilobular distribution	53 (100)	16 (100)	37 (100)	
Perilymphatic distribution	51 (96.2)	15 (93.8)	36 (97.3)	0.517
Lobular consolidation	33 (62.3)	11 (68.8)	22 (59.9)	0.522
Segmental consolidation	19 (35.8)	7 (43.8)	12 (32.4)	0.430
Peribronchial consolidation	32 (60.4)	10 (62.5)	22 (59.5)	0.835
Bronchiectasis	10 (18.9)	3 (18.8)	7 (18.9)	1.000
Classic miliary nodule	53 (100)	16 (100)	37 (100)	
Macro nodule	19 (35.8)	3 (18.8)	16 (43.2)	0.123
Cavity	12 (22.6)	0	12 (32.4)	0.010
Lymph node involvement				
Neck lymph node	7 (13.2)	1 (6.3)	6 (16.2)	0.661
Mediastinum	32 (60.4)	9 (56.3)	23 (62.2)	0.686
Axillar	2 (3.8)	0	2 (5.4)	1.000
Lymph node nature				
Necrosis	1 (1.9)	1 (6.3)	0	0.302
Calcification	8 (15.1)	4 (25.0)	4 (10.8)	0.224
Pleural effusion				0.998
Right	3 (5.7)	1 (6.3)	2 (5.4)	
Left	3 (5.7)	1 (6.3)	2 (5.4)	
Both	10 (18.9)	3 (18.8)	7 (18.9)	

Data are presented as the number (%).

Abbreviations: CT, computed tomography; TB, tuberculosis

Supplemental Figure 1. Representative chest CT images showing cavitary lesions in a culture-positive patient (a-b) and absence of cavitary lesions in a culture-negative patient (c-d).

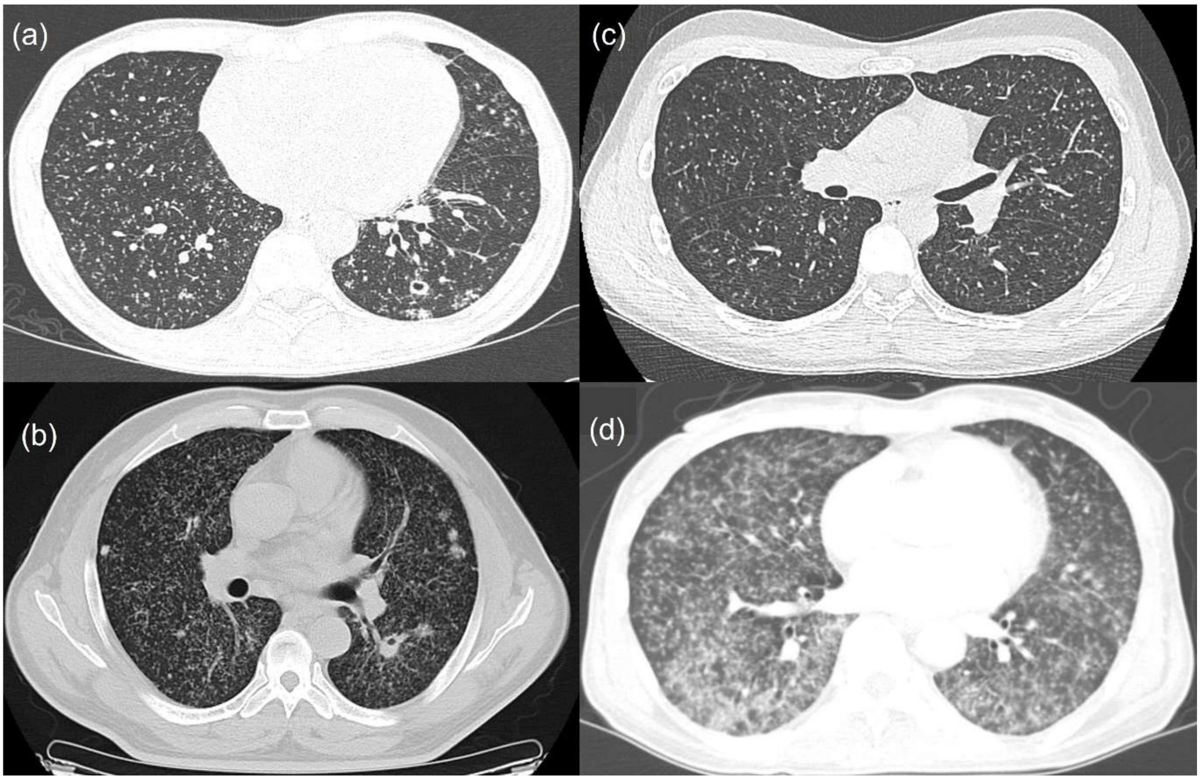

Supplement: Supplementary file 1 [file ijtldopen25-0579_supplementarydata1.pdf]
